# Toxicants and Teen Health: Pollutant Effects on Adolescent Thyroid Function

**Published:** 2008-06

**Authors:** Tanya Tillett

Persistent organic pollutants (POPs), as their name suggests, persist in the environment, contributing to the burden of toxicants threatening human health. The evidence to date strongly suggests these chemicals contribute to endocrine disruption, including altered thyroid function. Many studies have looked at health outcomes of POP exposure in infants and adults, but few have analyzed the effects in the age group in between: older children and adolescents. A new study now confirms a relationship in adolescents between altered thyroid function and prenatal exposure to certain POPs as well as to other common pollutants **[*EHP* 116:806–813; Schell et al.]**.

Researchers in the current study examined the effect of POPs including polychlorinated biphenyls (PCBs), hexachlorobenzene (HCB), and *p,p*′*-*DDE (a metabolite of DDT), as well as lead, mirex, and mercury, on thyroid function. Study participants included 232 mother–youth pairs of the Akwesasne Mohawk Nation, who lived near an area with a history of local environmental pollution from neighboring industrial complexes. Between 1996 and 2000, trained Mohawk staff collected fasting blood samples from the youths (aged 10–17 years) and provided material for serum level analyses of the six toxicants as well as cholesterol, triglycerides, and the thyroid hormones triiodothyronine (T_3_), thyroxine (T_4_), and thyroid-stimulating hormone (TSH). The mothers provided sociodemographic and breastfeeding history information.

Serum PCB levels were consistent with chronic exposure to multiple toxicants. Levels for 16 congeners—including 8 persistent (with long physiologic half-lives) and 8 nonpersistent—indicated both cumulative and recent exposures. Controlling for other toxicants, the investigators used multivariate regression analysis to examine the effects of PCB exposure on TSH, T_3_, and T_4_. They found that breastfed adolescents had higher levels of persistent PCBs and *p,p*′-DDE than non-breastfed adolescents. However, despite having lower levels of persistent PCBs, the non-breastfed adolescents displayed a significant positive relationship between persistent PCBs and TSH and a significant negative one between persistent PCBs and free T_4_, whereas breastfed adolescents did not.

The highest level of lead measured in the study population was less than half the Centers for Disease Control and Prevention action level of 10 mg/dL (lead was positively associated with T_3_). Mercury levels were well below the Environmental Protection Agency reference dose of 5.8 mg/L in all but one adolescent. More than 50% of the study population had mirex levels below the method detection limit of 0.02 ppb as well as a negative association of HCB with T_4_ levels.

Thyroid hormones are important because they regulate metabolic rate, growth, cognitive development, and many other important functions. These findings support the hypothesis that prenatal exposure to PCBs and other toxicants alters long-term thyroid function. Postnatal exposure cannot be excluded as an influence, but exposure from breast-feeding was not linked to an effect on thyroid hormones.

## Figures and Tables

**Figure:**
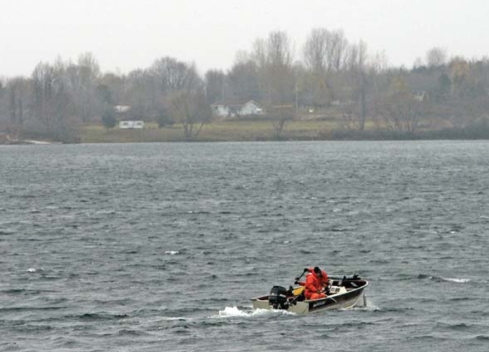
A fishing boat navigates the St. Lawrence River off the Akwesasne Mohawk Nation

